# Diseases of the Mastoid and Their Treatment

**Published:** 1889-06

**Authors:** T. J. Tyner

**Affiliations:** Austin, Texas


DANIEL'S

### TEXAS MEDICAL JOURNAL.

A Representative Organ of the Medical Profession, and an Exponent of Rational Medicine; devoted to the Organization, Advancement and Elevation of the Profession in Texas.

PUBLISHED MONTHLY.—SUBSCRIPTION $2.00 A YEAR.

Vol. IV. AUSTIN, JUNE, 1889. No. 12.

### ORIGINAL ARTICLES.

CONTRIBUTED EXCLUSIVELY TO THIS JOURNAL.

*The Articles in this Department are accepted and published with the understanding that we are not responsible for, nor do we indorse the views and opinions of the writers by so doing.*

### DISEASES OF THE MASTOID AND THEIR TREATMENT.

BY T. J. TYNER, M. D., AUSTIN, TEXAS.

Read at meeting of Austin District Medical Association and referred to Publishing Committee.

THE literature has been greatly enriched on this subject in the last few years, and it is now regarded as the most important question in aural surgery, for a life may depend upon early and correct diagnosis and judicious treatment.

### ETIOLOGY.

This is almost always found in disease of the middle ear, usually by extension through the mastroid antrum, owing to the obstruction to free drainage of the pent up secretions and diseased products. Inflammation frequently follows, particnlarly in children: measles, scarlet fever and diphtheria, but the middle ear is always first affected. It might be produced by traumatism.

It is claimed by some recent writers, that an acute primary inflammation of the mastoid cells is not infrequently met with.

PATHOLOGY.

The pathological changes are: congestion and inflammation of the mucous membrane lining the cells, periostitis of the outer surface, caries necrosis, etc.; of course, these charges will be determined by the character and duration.

SYMPTOMS AND DIAGNOSIS.

The symptoms are variable, and in many cases misleading, hence, the diagnosis is exceedingly difficult, particularly so in the incipiency, though, as a rule, as the disease is more advanced, the diagnosis is unmistakable. Pain, more or less severe, is nearly always present, but in some instances, is not referred to the affected spot. In such a case, with the pain very severe and persistent, the brain is most likely involved. Pain usually starts from the mastoid, and radiates towards the brow, vertex and occiput. In acute inflammation of the cellule, there is more or less redness and swelling over mastoid with pain, markedly so in periostitis or abscess of the outer surface. During the more acute paroxysm of pain, the auriel stands out farther from the head. This last symptom is regarded by some writers as pathognomonic of inflammation of the mastoid cavity.

With these symptoms, an inspection of the ear generally reveals the starting point of the trouble. The auditory canal is greatly swollen, or occluded by a polypus or other foreign substance. But if the canal should be free, the tympanum, if intact, is red, bulging and painful, or should there be an old perforation, it will be filled, or partially so, with granulations much thickened, and inadequate for the escape of pus, mucus, etc., from middle ear.

TREATMENT.

If the diagnosis is clear, the treatment is at once made manifest, but when this is not the case, there are so many points not yet understood, or insufficiently appreciated, that an accurate decision is impossible, even in the hands of the ablest expert. In the acute stage, with swelling, redness and pain, the inspection of the ear, and removal as far as possible of any obstruction to thorough drainage, is of the first importance. If the external auditory canal is free, paracentesis of typmpanum, or if an old perforation exist, it should be enlarged, granulation, etc., removed. Inflation with Politzer bag with the catheter or other methods. If the posterior wall of the external auditory canal bulges so as to nearly, or quite exclude the latter, frequent and prolonged douching with hot water, or it may be necessary to make a free incision, though this is exceedingly painful; or hot applications behind the ear. I usually order the entire ear covered; ice bags sometimes give prompt relief, when the hot applications have failed. Opiates and constitutional treatment are indicated. These measures judiciously carried out, together with a free incision behind the ear generally gives relief. This is called Wilde's incision, and as you probably know, is an incision down to the bone, about an inch and a half in length, parallel to the ear and a few lines behind it.

This incision is easily made, without danger, and should not be delayed too long. It frequently gives relief, even if pus is not found. Both in acute and chronic inflammation of the middle ear, abscesses may form on the outer surface of mastoid, having no connection with the cells. The incision would give relief at once and remove any suspicion as to disease of the cavity, unless there should be found a fistulous opening, which should always be searched for. Periostitis over mastoid, without disease of middle ear, is relieved by free incision, but if left to itself, may make its way into the external meatus. I have so much faith in Wilde's incision that I think, if it were made sufficiently early, the necessity of opening tbe mastoid cavity would be removed in a large number of cases.

INDICATIONS FOR OPENING THE MASTOID CAVITY.

With all the progress made in aural surgery in the last ten or fifteen years, the question: What are the conditions under which it should be opened? is still unanswered. Were I asked the question I would say, not until the measures, outlined above, after a sufficient trial, have failed, and even then I should be inclined to wait, unless the demand for interference were imperative.

Could we be positively certain that the pressure of pent up secretions gave rise to cerebral complications, all cause for hesitation would be removed, but on the contrary, we know that the brain may be implicated rather from transmission by continuity, and the anastomosis of vessels. In acute disease of middle ear there may be great swelling and pain over mastoid, and yet the dura mater become involved, while the mastoid cells remain comparatively free from complications. In the case of a young lady, about a year ago, at my evening visit the symptoms were so urgent, I decided to operate the next morning; would have done so then, but it was after dark and I had no one to assist me. The next morning I found her bright and happy. During the night a free escape of pus from middle ear had taken place, with perfect and permanent relief. I mention this case as an illustration of many others. Some authorities advise the operation when symptoms point to an accumulation of pus, even if the bone appears healthy. There could be no argument against such a position, if there were any symptom or set of symptoms positively pointing to such accumulation, but unfortunately there are no such.

It is not my desire to be understood as opposing the operation, but to show the difficulties attending the indications for the operation. There are many cases in which there are no such difficulties, aud others again, where the symptoms are so perilous that no choice is left but to operate.

### METHOD OF OPERATING.

The operation has such a unique history, I know you will pardon a few lines to it. It was done first by Petit, who died in 1750. Also by Jasser, a Prussian military surgeon, who was probably unaware of the labors of his distinguished French confrere. Their contemporaries and immediate successors did not understand the indications for the operation, and it was resorted to for all forms of deafness; and Baron von Berger, physician to the king of Denmark, allowed himself operated on for deafness and noises in the ear, which was followed by fever and dilirium and death on the eleventh day, From that time on the operation became obsolete and was mentioned only as a curiosity of history, until revived by Ferget in 1849.

Opening the cavity is best done with chisel and mallet, though the trephine, drills, gimlet, etc., are used by many With the chisel every step can be inspected. This advantage alone should give it precedence over all others. A vertical incision is to be made in the soft parts down to the bone, a few lines behind the ear, with its center on a level with the center of the auditory canal, and from this point a second incision should be made, directly backwards, one inch long, the soft parts dissected up and the periosteum raised. The bone should be chipped off in thin layers as close to the posterior wall of the meatus as possible. Five-eighths of an inch is as deep as it is prudent to go, according to most authors, as there is danger of wounding the facial nerve, lenticular canals and other structures. Frequent examinations should be made as to the character of the tissues and as to the position in relation to the auditory canal. The insertion of a large blunt probe in the canal, is a very convenient way to guard against the danger of a mistaken course, as it determines the relative position of the opening to the canal. When the cells have been entered, their candition and character will indicate the correct measures to adopt as regards drainage, etc. The subsequent treatment, as a rule, requires great care and close attention for weeks and, perhaps, months.

